# The Remapping of Peripersonal Space in a Real but Not in a Virtual Environment

**DOI:** 10.3390/brainsci12091125

**Published:** 2022-08-24

**Authors:** Francesca Ferroni, Vittorio Gallese, Agata Marta Soccini, Nunzio Langiulli, Francesca Rastelli, Donato Ferri, Francesco Bianchi, Martina Ardizzi

**Affiliations:** 1 Unit of Neuroscience, Department of Medicine and Surgery, University of Parma, 43126 Parma, Italy; 2 Computer Science Department, University of Torino, 10124 Turin, Italy; 3 EY Advisory, 00187 Rome, Italy

**Keywords:** action, multisensory integration, plasticity, space, tool use, virtual reality

## Abstract

One of the most surprising features of our brain is the fact that it is extremely plastic. Among the various plastic processes supported by our brain, there is the neural representation of the space surrounding our body, the peripersonal space (PPS). The effects of real-world tool use on the PPS are well known in cognitive neuroscience, but little is still known whether similar mechanisms also govern virtual tool use. To this purpose, the present study investigated the plasticity of the PPS before and after a real (Experiment 1) or virtual motor training with a tool (Experiment 2). The results show the expansion of the PPS only following real-world tool use but not virtual use, highlighting how the two types of training potentially rely on different processes. This study enriches the current state of the art on the plasticity of PPS in real and virtual environments. We discuss our data with respect to the relevance for the development of effective immersive environment for trainings, learning and rehabilitation.

## 1. Introduction

Through evolution, the brain has functionally developed a modular representation of space creating an imaginary sector immediately surrounding our body, known as peripersonal space (PPS) [1,2,3]. This region of space is a multisensory space where visual or auditory inputs related to the environment are integrated with tactile and proprioceptive information concerning specific body parts [1,4,5,6]. This spatial representation remaps plastically after different experiences, e.g., it expands after tool use [7,8,9,10,11], it contracts after immobilization [12], it blurs after sensory deprivation [13], or it is modulated by the type of social context [14,15]. Several studies have described the changes in the representation of PPS after interacting with physical tools [16,17,18]. However, we often interact also with remote tools to control computers, such as a keyboard, computer mouse or joysticks. As an example of the variety of applications, more and more surgeons interact daily with robotic devices that allow them to perform complex surgical procedures in a more accurate and efficient manner without being in direct contact with the patient [19,20,21]. In these contexts, our relationships with objects/people in the space surrounding our body–PPS–are affected. Indeed, although still few in number, some studies have started to investigate how PPS can be influenced by remote interactions. It has been in fact demonstrated that PPS is shaped by remote interaction mediated by the computer mouse [22] or surgical robots [23,24]. Specifically, Sengül et al. [24] have demonstrated that the active use of a robotic tool changes the integration of multisensory information, assessed by means of the cross-modal congruency task in PPS, comparable with the remapping of PPS during real-world tool use. Moreover, Bassolino and colleagues [22] showed that the space where a pointing tool (i.e., a computer mouse) was actually held, close to hand, was extended to the space where it operated (i.e., the computer screen), even though these spaces were not physically connected. Alongside increasingly cutting-edge technologies that integrate real-objects and remote virtual interactions, immersive virtual technologies are finding widespread applications in our hybrid (i.e., real–virtual) world. Today, the virtual reality environment is a valid technology applied to several activities, including neuropsychological rehabilitation [25]; psychopathological exposure therapy [26]; and military, educational and surgical training [27,28]. In these virtual immersive environments, people have the ability to move around in the space with the potential to manipulate or interact with objects much as they could in the real world. Despite all these technical advances in the field of virtual immersive technologies and a recent growing interest of neuroscience in virtual environments, e.g., [15,29,30,31,32,33], little attention has been paid to the neural and behavioral mechanisms underlying the interactions with these devices and the consequent remapping of the physical milieu surrounding our body. Thus, taking into account the potentially relevant implications on our current world and the physical space in which we act every day, it is relevant to investigate what happens during a virtual interaction with a tool in one’s own PPS. To fill this gap, we have investigated the plasticity of PPS after two different motor training in a real (Experiment 1) and a virtual immersive environment (Experiment 2). Based on previous observations [30,34], in both tasks, we assess the extension of PPS in an immersive virtual reality environment. With respect to previous studies in this field [35,36] that adopted not widely used tasks as accurate proxies for measuring PPS, we decided to use a multisensory task to measure PPS in order to have more generalizable results with the current literature in this field. Moreover, we adopted a within-subjects experimental design to compare the performances of participants during real (i.e, real-world tool use) and virtual (virtual-world tool use) trainings. Taking into account evidence from several previous studies on PPS remapping after tool use [7,8,11,22], we expected (i) to confirm the validity of the visuo-tactile PPS task [29,30] in detecting PPS boundaries; (ii) to find a PPS expansion after the real-world tool use, accordingly with the current wide literature e.g., [7,8,37]; (iii) to detect a similar expansion, or slightly smaller, after the virtual motor training than that observed during the real training.

## 2. Materials and Methods

A total of 22 participants took part in this study (9 males, M = 24.57 years, SE = 5.24). Participants performed both Experiment 1 and Experiment 2 (within subjects design), administered in a balanced manner among participants. Participants’ handedness was assessed by the Edinburgh handedness inventory [38] (M = 0.83, SE = 0.18). All participants had normal touch and normal or corrected-to-normal vision. The study was approved by the Local Ethical Committee (AVEN) and was carried out in accordance with the Declaration of Helsinki (1964 and subsequent amendments).

### 2.1. Procedure

The experimental procedure, identical for Experiments 1 and 2, consisted of three sessions all carried out on the same day. First, participants performed the visuo-tactile Peripersonal Space task (Session 1) in order to measure the individual PPS at baseline. After this session, they took part in Session 2 (i.e., training phase, different for Experiment 1 and 2; see below). Lastly, participants were submitted again to the PPS task (Session 3) in order to measure PPS boundaries after the Session 2.

#### 2.1.1. Session 1 and 3

The location of participants’ PPS boundary was measured with the adapted version of the visuo-tactile task widely adopted to measure PPS extent [30,39,40,41,42,43,44] implemented by our group using Unity3D 2020 for Meta Quest 2. Participants were seated behind a table and were asked to wear the head-mounted display while holding the controllers in both hands. They were instructed to ignore the presentation of the approaching or receding visual stimuli, travelling at the velocity of 75 cm/s, and respond as fast as possible to the vibrotactile stimulation, administered on the right controller, by pressing the button on the left controller. The visual stimulus was a tridimensional virtual red ball, 6.5 cm in diameter, looming toward the right hand of the participant. The ball travelled in virtual space from far to near or vice versa in the case of receding stimuli. Tactile stimuli of 10 ms of duration were delivered at 5 different temporal delays from the onset of the looming and receding visual stimuli (after 2165, 1732, 1299, 866, and 433 ms), resulting in 5 different distances from the body (D1–D5, ranging from 37.12 to 167.03 cm from the participant, in 32.5 cm intervals), following the procedure adopted by Masson and colleagues [29]. Trials were equally divided into two blocks for a total of 220 trials, lasting about 8 min each. Each trial was repeated if participants failed to respond to the tactile target. For a detailed description of the procedure, please refer to Supplementary Materials (Section 1).

#### 2.1.2. Session 2

**Figure 1:**
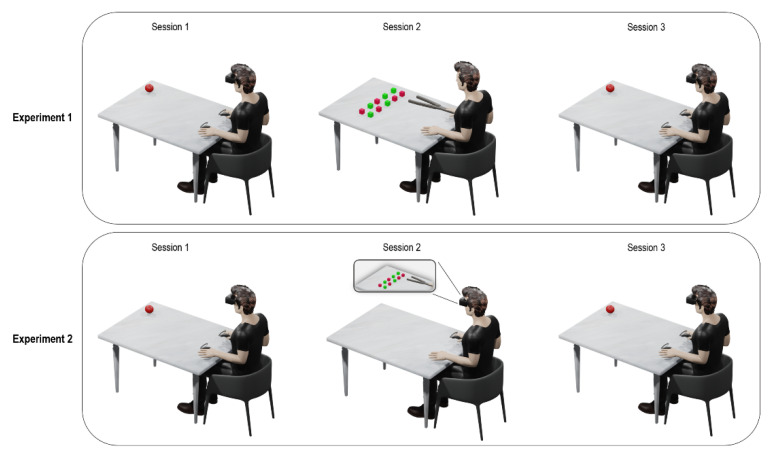
Graphical representation of the experimental procedure. For both experiments, Session 1 and Session 3 show the experimental setting of the visuo-tactile Peripersonal Space (PPS) task. Session 2 shows the qualitative representation of the training phase for both Experiments 1 and 2.

Session 2 differed between the two experiments. Specifically, in Experiment 1, during Session 2 participants were instructed to move 50 small colored objects (green and red), placed on two marked areas of the table, in the far space (85 cm from participants’ chest) [8]. Participants sat along the short side of the table and were requested to use a tool to grab and move one object at a time across the two areas. All objects were moved from one marked area to the other and then repositioned on the initial area for a total of 100 movements (Figure 1). In Experiment 2, participants performed the virtual version of the motor training adopted in our previous study [8], described above. Participants were instructed to move 50 small colored virtual objects (green and red), placed on two marked areas of the virtual table in far space, using a virtual 75 cm-long garbage clamp held pressing a button of the right controller, as in Experiment 1. Participants had to wear a pair of white surgical gloves in order to promote a sense of embodiment with the virtual white hands, as we did not use an avatar with humanoid appearance and with phenotypic characteristics of the participants’ real hand. In both experiments, Session 2 lasted around 10 min (Figure 1).

## 3. Data analysis and Results

### 3.1. Multisensory Tactile RTs

We first performed an analysis of variance (ANOVA) to check the different modulations of the looming compared with receding stimuli on tactile reaction times (RTs) independent of condition (Session 1, Session 3) or experiment (Experiment 1, Experiment 2) at different distances (D1, D2, D3, D4, D5). Specifically, data were entered in a repeated-measures ANOVA with two within-subject factors, Visual stimuli (Looming, Receding) and Distance (D1, D2, D3, D4, D5). For RT measurement, please consult the Supplementary Materials (Section 2). The ANOVA showed a significant effect of visual stimuli (F_(1,80)_ = 14.80, *p* < 0.001, η^2^_p_ = 0.42). Indeed, it has been repeatedly shown that the present task is especially sensitive to approaching as compared with receding stimuli [7,14,39,42,45]; therefore, we here focused on results concerning the Looming visual stimuli only (Receding visual stimuli data are reported in Supplementary Table S1), as in previous studies on PPS, e.g., [42]. The function describing the relationship between tactile RTs and the perceived position of the visual stimuli in space showed that tactile RTs progressively sped up as the perceived visual stimuli’s distance from the body decreased, as expected. Specifically, the ANOVA conducted on Looming stimuli only revealed a significant main effect of Distance (F_(1,4)_ = 11.10, *p* < 0.001, η^2^_p_ = 0.36). Newman–Keuls post hoc tests showed that RTs at D1 (when the visual stimuli were perceived far from the body; M = 560.53 ms, SE = 20.40) and D2 (M = 535.31 ms, SE = 12.91) were significantly slower compared with RTs at D3 (when the visual stimuli were perceived close to the body; M = 515.53 ms, SE = 12.23), D4 (M = 499.28 ms, SE = 12.15) and D5 (M = 498.48, SE = 12.70; all *p*_s_ < 0.03). This was considered a preliminary step in order to proceed to considering only Looming stimuli as experimental variables.

### 3.2. Unisensory Tactile RTs

Additionally, an ANOVA across sensory modalities was carried out to confirm that multisensory looming trials (regardless of distance) were faster than unisensory tactile trials, and thus, as expected, visual presentations facilitated tactile responses e.g., [46]. Specifically, we performed a repeated-measures ANOVA on Looming and Unisensory RTs to tactile targets measured across sensory modalities to confirm the multisensory facilitation effect independently from Distance (D1, D2, D3, D4, D5), Condition (Session 1, Session 3) or Experiment (Experiment 1, Experiment 2). Thus, RTs were entered in a repeated-measures ANOVA with sensory modality (multisensory, unisensory) as the within-subjects factor. The ANOVA showed a significant main effect of sensory modality (F _(1,20)_ = 55.77, *p* < 0.001, η^2^_p_ = 0.74), showing that multisensory RTs (multisensory: M = 521.83, SE = 12.58) were faster than the unisensory tactile ones (unisensory: M = 604.11, SE = 16.64), demonstrating a clear multisensory facilitation effect in line with previous studies e.g., [9,46].

### 3.3. Peripersonal Space Estimation

**Figure 2:**
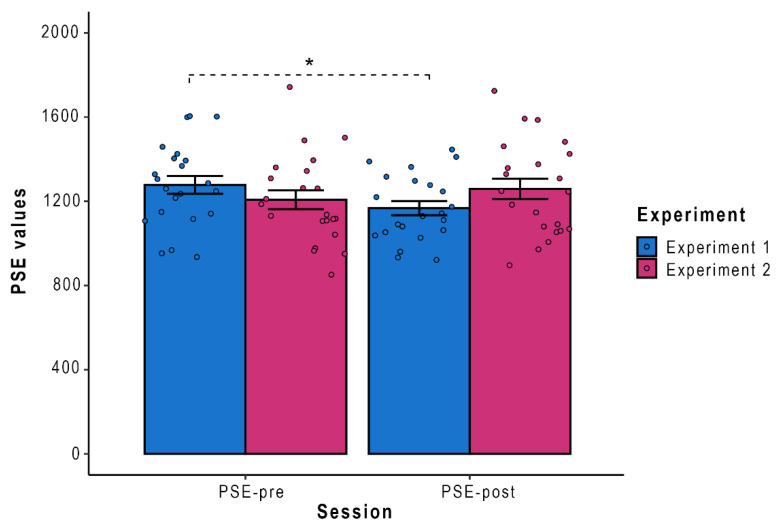
Point of subjective equality (PSE) values measured in Session 1 and Session 3, for both Experiments. Error bars depicted SE; * = *p* < 0.05.

Lastly, to estimate the individual boundary of PPS, PSE (point of subjective equality) of the psychometric function describing visuo-tactile RTs as a function of visuo-tactile distance was measured via the Spearman–Karber (SK) method [47,48] in line with recent studies on PPS [9,29,46]. For more specific details about the implemented procedure, please refer to the Supplementary Materials (Section 2). PSE values estimated using Looming RTs in Session 1 (PSE-pre) and in Session 3 (PSE-post) were entered into ANOVA with Condition (PSE-pre, PSE-post) and Experiment (Experiment 1, Experiment 2) as within-subjects factors. Results showed a significant interaction Experiment by Condition (F _(1,21)_ = 9.37, *p* = 0.005, η^2^_p_ = 0.31). Newman–Keuls post-hoc carried out on the significant interaction Experiment by Condition revealed that PSE-pre values (M = 1277.50 ms, SE = 42.31) were significantly higher than the PSE-post ones (M = 1167.63 ms, SE = 33.60, *p* = 0.03) only for the Real Training (Figure 2), thus revealing a peripersonal space expansion only after the real-world tool use (Experiment 1). No differences were found between PSE-pre and PSE-post values after the virtual-world tool use (Experiment 2) or in any other comparison (all *p*_s_ > 0.08).

### 3.4. Slopes Estimation

The slope’s values (hereafter DL, difference limen, estimated via the SK method) measured in Session 1 (DL-pre) and in Session 3 (DL-post) were entered into ANOVA with Condition (DL-pre, DL-post) and Experiment (Experiment 1, Experiment 2) as within-subjects factors. No significant results were found (all p_s_ > 0.09).

For all the analyses, whenever appropriate, significant differences were explored performing Newman–Keuls post-hoc comparison. Partial eta-squared (η^2^_p_) was calculated as effect size measure.

## 4. Discussion

The present study investigated the plasticity of PPS after a real (Experiment 1) and a virtual motor training session (Experiment 2) executed with a tool in the extrapersonal space. To reach this goal, participants performed a visuo-tactile interaction task, e.g., [15,29] to identify the distance at which an approaching visual stimulus speeded up tactile processing as a proxy for the boundary of PPS, both before and after the two different training sessions with a tool. Our results confirm the validity of the visuo-tactile PPS task [30,34]. Indeed, independently from Experiments 1 and 2, we showed that a virtual approaching visual stimulus sped up participants’ reaction time to match a tactile stimulation on their body at Session 1. This visuo-tactile interaction effect depended on the distance between the virtual stimulus and the body of the participant, as a significant facilitation emerged specifically when the virtual object was closer than a certain distance, which can be measured as a proxy of the location of the boundary of the individual’s PPS, e.g., [15,30]. Importantly, we found a PPS expansion only after the real-world tool use (Experiment 1), as shown by the lower PSE-post values than the PSE-pre ones only in the case of Experiment 1. Differently, and contrary to our expectations, an effect of virtual tool use in the remapping of PPS was not found, as no significant change was pointed out between PSE-pre and -post values in the case of Experiment 2.

Today, we live in an unprecedented condition in which the space around our bodies can abstract from physical contingency and might have a virtual immanence. The data of the present work show that under the same minimal conditions (the appearance and type of interactions in the two experiments were in fact the same), the virtual environment is less effective than the real one in stimulating the plasticity of PPS. Multiple factors, which must be taken into account when developing immersive virtual environments, could explain this difference.

Firstly, participants were not familiar with the use of the virtual tool. Indeed, no preliminary familiarization phase with the virtual tool was carried out. Despite it is well known that the boundaries of PPS enlarge even after an interaction with a real unfamiliar object [49], it is possible that the use of a virtual unfamiliar tool requires an extra-familiarization phase to elicit a PPS expansion. Knowing that, the higher the level of practice with a real tool, the longer the extension of PPS [11,50], it is possible that a virtual training relies on different temporal dynamics than those of the real training, requiring a longer time to elicit the expansion.

Secondly, we did not use an avatar with humanoid appearance and with phenotypic characteristics of the participants’ body. Participants simply saw virtual white hands moving in synchrony with their own hands while wearing white surgical gloves. In agreement with studies showing that action self-attribution in virtual environment resulted from an interaction between bottom-up and top-down processes e.g., [51], the here implemented dynamic visual congruency was not enough to induce the integration of the virtual tool into the participants’ body representation. Considering also previous studies highlighting the impact of the bodily self on the encoding of virtual environment e.g., [52,53,54], to develop effective virtual immersive environment the inclusion of an avatar body in a first-person perspective should be considered.

Lastly, in the present setup, a tactile vibration was delivered by the controller when participants grasped the virtual object with the virtual pliers. However, other proprioceptive and sensory feedbacks resulting from the movement of the real objects (e.g., weight changes at the end of the clamp) were missing in the case of virtual objects. Often surgeons and robotics researchers report this lack of sensory feedback, thus highlighting a significant limitation in robot-assisted minimally invasive surgery [55]. To date, the issue of haptic and proprioceptive feedbacks in robotic surgery systems is still a major technological limitation that does not allow surgeons to feel the interaction between the tool and the anatomy, operating with obscured sensory feedback [56]. Providing sensory feedback in virtual training can produce a strong change in PPS representation and a relevant remapping of visuo-tactile integration, not just for virtual tool use training but also in action observation task [36].

At first glance, it is impossible to define which of these hypothesized factors might be the most plausible one. To our knowledge, this is the first study in which the expansion of the PPS is tested in a virtual immersive environment, by adopting a multisensory interaction task as a proxy for the PPS. As a benchmark, we can certainly confirm that the minimum conditions proposed here (and currently available to the common virtual reality devices) are not sufficient to induce PPS plasticity. It is of interest to emphasize how these conclusions can lead to improvements in both neuroscientific research and the application of virtual reality [57]. Indeed, on the one hand, the use of virtual settings in neuroscientific research can shed light on the processes required to induce plasticity in the PPS, which to date have been little investigated [42]. By individually modifying the factors evidenced here (i.e., familiarity, body presence and sensory feedback) in future studies, it will be possible to determine what are the necessary and sufficient conditions to induce a PPS expansion in virtual immersive environments. There is no doubt that virtual environment is the only context in which it is possible to manipulate each of the aforementioned factors individually. On the other hand, virtual technologies are finding widespread applications in our hybrid-world and they are progressively more popular in various fields (e.g., information services, video-games, people services), especially now that spatial computing and the metaverse are promising to be the next standard paradigm of the internet and therefore become pervasive in everyday life. Understanding what are the mandatory features that our brain requires to allow a virtual environment to induce plastic change can greatly enhance the performativity of the designed environments. Indeed, although much is already known about the neural basis underlying the representation of PPS e.g., [58,59,60,61], the neural mechanisms underlying the PPS plasticity elicited in real and virtual environments still remain to be well understood. The resulting increased concreteness of virtual environments may turn out to be of pivotal importance for the design of future learning, training, and rehabilitative protocols [62,63].

## 5. Conclusions

The present study provides the first evidence of the expansion of the PPS only following a real world tool use but not a virtual one, highlighting how the two types of training potentially rely on different processes. The unity of our bodily experiences with respect to an ever-changing and evolving world is a fundamental condition of human interaction with the external environment, even when the environment is virtual. Indeed, the way in which the environment offers more and more variation affects the impact of the space (around us) on humans and vice versa. Thus, in order to maintain this mutual interaction, it is necessary to investigate the virtual environment from a neuroscientific perspective so that it is truly a space for (inter)-action.

## Data Availability

The data presented in this study are available on request from the corresponding author.

## Supplementary Materials

The following supporting information can be downloaded at: https://www.mdpi.com/article/10.3390/brainsci12091125/s1, Table S1: Data of Receding RTs at five different distances [64,65].
