# Supplementary material for: The Remapping of Peripersonal Space in a Real but Not in a Virtual Environment
Source: Brain Sci. 2022 Aug 24;12(9):1125. doi: 10.3390/brainsci12091125 (PMC9497134; doi:10.3390/brainsci12091125)
Supplement: Supplementary file 1 [file brainsci-12-01125-s001.zip › brainsci-1863736-supplementary.pdf]

1. Apparatus and Procedure

The size of the peripersonal space was assessed with a well-established visuo-tactile interaction task [42,43]. Participants were informed that they would have felt a vibration on their right hand and seen a virtual ball. They were also told that the virtual ball was task irrelevant, and instead asked to respond as quickly as possible to the tactile vibration by pressing a button on the left controller. The logic of the PPS task is as follows: PPS neurons respond both to touch and the visual stimuli presented in the near space [3-5]. Thus, when visual stimuli are far, there should be no multisensory interaction. But when visual stimuli are presented near, within the receptive fields of visuo-tactile neurons, visuo-tactile multisensory interactions should speed reaction times to touch [e.g., 7,42,43]. Thus, we presented tactile stimulation while a visual stimulus was at different distances, and we aimed to determine the furthest distance from the body at which a visual stimulus significantly speeds up tactile processing. That is, the distance at which visuo-tactile RTs are significantly faster than RTs to unimodal tactile stimulation is a proxy for the PPS boundary [30,42,43].
Each trial in the task was 2660 ms long and on each trial, tactile stimulation (vibration) was administered at one of five different temporal delays from the onset of the trial and the onset of the visual stimuli looming or receiding toward participants (after 2165, 1732, 1299, 866, and 433 ms). We included three types of trials presented in randomized order—only tactile trials, visuo-tactile trials, and catch trials. 60.60% of the trials were experimental bimodal visuo-tactile trials, in which the tactile stimulus was delivered in combination with the approaching or receding visual stimulus (as described above). 30.30% of trials were unimodal tactile only trials, in which the tactile stimulus was delivered in the absence of the visual stimulus. These trials are considered baseline trials and are used to show the bimodal facilitation effect on RTs to tactile stimuli. The only tactile trials are used to control for individual differences in RTs to tactile stimuli. In both the unimodal and bimodal trials, the tactile stimulus was delivered at one of the five distance points (D1–D5) in randomized order, to prevent expectancy effects. Lastly, 9.10% of trials were catch trials in which the visual stimuli were presented and no tactile stimulus was delivered. Catch trials necessitate withholding the response and thus ensure that participants are attentive to the task. Further, they mitigate the entrainment of an automatic motor response and an expectancy effect— that tactile stimulus is more likely to occur the longer it has been since trial onset ([65-66]. In total the task consisted of 220 trials: 15 visuo-tactile trials per distance (75 total for looming-tactile trials; 75 total for receding-tactile trials); 10 tactile trials per distance (40 total) and 15 catch trials per visual stimuli (15 looming and 15 receding catch trials). A fixation cross was presented at the beginning of the task and was offset once a key was pressed to begin trials. The total duration of the task was approximately 16 min.

2. Spearman-Karber method procedure

The 10% trimmed means of participants' reaction times (RTs) obtained in response to the tactile stimulus administered at the five distances (D1, D2, D3, D4, D5) were used to estimate the point of subjecting equality (PSE), as a measure of location (or central point estimation) and difference limen (DL), as a measure of steepness, of the psychometric function underlying the data [49]. To estimate these parameters without any specific assumptions about the function family of the underlying psychometric function, we used a non-parametric approach, the Spearman-Kärber method [48,49], as done also by other previous studies [29,47,9]. All codes are available upon reasonable request made to the corresponding author F.F and to N.L.

Supplementary Figures

Supplementary Table S1. Data of Receding RTs at five different distances.

D1	D2	D3	D4	D5
M= 567.90, SE= 18.64	M= 537.43, SE= 13.58	M= 529.16, SE= 16.16	M= 536.90, SE= 15.05	M= 514.41, SE= 10.67
